# Experimental study with nursing staff related to the knowledge about pressure ulcers^1^

**DOI:** 10.1590/1518-8345.1134.2831

**Published:** 2016-11-21

**Authors:** Miriam Viviane Baron, Cézane Priscila Reuter, Miria Suzana Burgos, Veniria Cavalli, Cristine Brandenburg, Suzane Beatriz Frantz Krug

**Affiliations:** 2 MSc, Researcher, Universidade Federal do Rio Grande do Sul, Porto Alegre, RS, Brazil; 3 MSc, Assistant Professor, Universidade de Santa Cruz do Sul, Santa Cruz do Sul, RS, Brazil; 4 PhD, Full Professor, Universidade de Santa Cruz do Sul, Santa Cruz do Sul, RS, Brazil; 5 Intensive care specialist, RN, Hospital Santa Cruz, Santa Cruz do Sul, RS, Brazil; 6 MSc, Researcher, Universidade Federal do Ceará, Fortaleza, CE, Brazil

**Keywords:** Pressure Ulcer, Education, Continuing, Intensive Care Units

## Abstract

**Objective:**

to compare the scores of knowledge in teams participating or not participating in educational interventions about pressure ulcers.

**Method:**

a quantitative study with experimental design. Data were collected through a validated questionnaire. The study included 71 individuals, including nurses and nursing technicians from three intensive care units, divided into intervention group and control group. Data analysis considered the scores of the groups in the moment before and after intervention. To check the average rate of correct answers, we calculated the mean and standard deviation. We carried out the Mann-Whitney test for analysis of two independent samples, and the Wilcoxon test for related samples.

**Results:**

The mean percentage of correct answers, at the baseline was 74.1% (SD = 26.4) in the intervention group and 76.0% (SD = 22.9) in the control group and post time -intervention, was 87.8% (SD = 18.8) in the group receiving educational intervention, considering that in the control group it was 79.1% (SD = 22.2). The group that participated in educational interventions did not reach the proper average of 90% correct answers for the test.

**Conclusion:**

educational interventions on staging, evaluation and prevention of pressure ulcers contributed significantly to the increase of correct responses score in the knowledge test of the intervention group and improved their knowledge on the subject.

## Introduction

In the complex environment of the Intensive Care Unit (ICU) critical health condition patients, who are comatose, sedated or unconscious are hospitalized. Several of them are intubated and need devices to maintain stable physiological functions. These patients can be bedridden for long periods and, therefore, are more vulnerable to adverse events related to health care, such as pressure ulcer (PU)^1^ ^-^ ^2^.

The PUs have a negative impact on the quality of life of patients, hindering functional recovery, often causing pain and suffering, leading to the development of serious infections associated with prolonged hospitalization, sepsis and mortality^3^ ^-^ ^6^. Moreover, the PUs expand the workload of healthcare staff and contribute to expensive costs for health services^7^. Because of this problem, the health insurance of the *Center for Medicare and Medicaid Services (CMS)* - EUA - no longer reimburses the excess cost of acquired PUs during hospitalization^8^, and this may be a tendency of health plans in Brazil.

Incidence rates and prevalence of PU in Brazilian hospitals remain high while there are timid initiatives to prevent this injury. A study conducted in a public hospital in the Federal District indicated prevalence of PU in 87.5% of patients in the trauma ICU and 50% in the general ICU, and in that same hospital, the incidence of PU was found to be 41.6% in the trauma ICU and 33.3% in the general ICU^3^. Other studies in ICU in Brazil showed the incidence of 41.0% and 25.7% respectively of PU, in São Paulo and Rio Grande do Sul^9^ ^-^ ^10^. In the international context, the United States PU prevalence rates acquired in ICUs vary from 8.8% to 12.1% depending on the type of ICU, and are considered by the authors as high rates in the US^11^. Countries in the Asian and European continents, show incidence rates of 11.2% in Japan and 14.9% in Germany^12^ ^-^ ^13^.

The presence of PU is a classic negative indicator of care quality^14^. European and US guidelines, such as the *European Pressure Ulcer Advisory Panel (EPUAP)* and the *National Pressure Ulcer Advisory Panel (NPUAP)*, provide appropriate practical recommendations, based on scientific evidence to treat and prevent the PUs^15^. In Brazil, there are no regulations and guidelines for the prevention of PU. However, European and US guidelines are prepared for all countries and are available for the Brazilian reality^9^.

PU is a problem of multifactorial origin and involves the whole multidisciplinary team in ICUs, especially the nursing teams, offering continuous care to patients^3^. In this regard, a routine of learning about PU should integrate the continuing education programs (EC - Continued Education Program ) in hospitals, aiming to contribute to timely and safe care in ICUs^16^. A study in Brazil shows that measuring the level of knowledge about the PU in nursing teams helps to identify the shortcomings of such knowledge and helps to guide the strategies for dissemination and adoption of preventive measures^14^. The implementation of risk assessment protocols and PU prevention correlates with a sharp decline in the incidence of these lesions^9^.

Seeking to contribute to the advancement of knowledge in this field, this research was conducted with nursing teams of three hospital ICUs of Rio Grande do Sul, aiming to compare the scores of knowledge about PU of the team who participated in the educational intervention with the scores of the team that did not participate in the educational intervention.

## Method

A quantitative study with experimental design, developed in three different ICUs in general hospitals of the region *Vales do Rio Pardo e Jacuí* in Rio Grande do Sul. One is a large hospital with ten beds in the ICU and two medium-sized hospitals, one with ten beds and other with seven beds. The survey was conducted from May to August 2014. It was approved by the Ethics Committee of the University of Santa Cruz do Sul number 639.428 and CAAE number 19329014.0.0000.5343. The professional staff of the acting nursing teams in adult ICUs was composed of 12 nurses and 62 nursing technicians, a total of 74 individuals from three different hospitals. It was decided to interview all acting subjects in ICUs who were not on vacation at the time of application of the initial survey questionnaire.

For the definition of the groups, three tickets with the name of ICUs were drawn and a random drawing was conducted. The first two drawn ICUs were defined as the intervention group and participated in educational interventions. The last ICU drawn was defined as the control group and did not participate in educational interventions. As inclusion criteria, all the subjects who agreed to participate and signed the Informed Consent Form (ICF) were considered. As exclusion criteria, employees who were on vacation in the initial questionnaire application stage were considered. Questionnaires of subjects who expressed a knowledge score equal to or greater than 90% in the first evaluation test were not considered for the study. For the final data of the intervention group, subjects who had frequency lower than 75% in educational interventions were excluded too, but these situations did not occur.

To obtain the data, we used an instrument composed of items related to socio-demographic data and a knowledge test on PU. For this, we used the Pieper test, validated and adapted in a previous study in Brazil^17^. The test is validated for use with nursing professionals in the ICU. This test is based on recommendations proposed by international guidelines and is comprised of 41 true or false statements with eight questions about staging and evaluation of PU and 33 on the prevention of PU. For each proposal, the participant must select an answer, considering the true (T), false (F) and do not know (DNK) options. The correct responses correspond to the true statements answered as T or false answered as F. One point was awarded for each correct answer. For wrong answers or those answered as "do not know", the assigned score was zero. The total score of the knowledge test corresponded to the sum of all the correct answers. According to the test authors, the participant's knowledge level was considered adequate when reached 90% or higher on the test, and the item was considered known when 90% or more of the participants answer correctly^18^. A pilot study was carried out for instrument verification, with respect to its presentation and understanding, as well as measuring the time to fill it.

Two main researchers carried out data collection and educational interventions. The instrument containing the questionnaire on socio-demographic data and knowledge test, was distributed to the subjects who answered individually and immediately during working hours and turning it in to researchers in an envelope without identification. The educational interventions with the teams of the intervention group were held once a week for ten weeks, thus totaling ten meetings of one hour. The educational interventions were performed in the ICU rooms as workshops. The interventions used resources such as slides in P*ower Point*, group discussions, positioning practice and printed slides handouts. The main topic discussed was the general description and recommendations on prevention of PU, based on international guidelines *EPUAP/NPUAP* ^15^. After the closing of educational interventions, the knowledge test was applied for both the intervention group and for the control group. After the end of the survey, an educational *workshop* on prevention of PU was conducted with the control group.

Data were transcribed with independent double typing, using The EpiInfo 7.0 program and then analyzed in the program *Statistical Package for Social Science (SPSS)*, version 20.0. To test the distribution of data, we used the Kolmogorov-Smirnov test. As data presented a not normal distribution, non-parametric tests were used. The variables related to socio-demographic characteristics were summarized and shown descriptively through distribution of frequencies and percentages. The scores of correct answers are shown as means and standard deviation. We performed the sum of questions, in which the participants obtained 90% or more of correct answers in the two periods. The chi-square test was used to assess relationships between socio-demographic variables and knowledge as well as to verify the association of correct scores at both times. For the verification of possible differences in the scores of correct answers between the groups, we applied the Mann-Whitney test and, between the two period, pre and post, the Wilcoxon test. In all statistical tests, the significance level was α ?λτ;0.05.

## Results

Of the total of 74 people that participated in the research, there was a breakdown of 71 professionals (there were 4.9% of losses resulting from refusal to participate in research and vacation), 50 of which belonged to the intervention group and 21 to the control group. Among these, 12 (16.9%) were nurses and 59 (83.1%) nursing technicians. During the period of educational interventions there were no losses in the intervention group. There were losses in the final collection of questionnaire data in the control group, which was restricted to eight (38.0%) people, due to vacation, days off and layoffs. The distribution of participants according to socio-demographic characteristics is found in Table 1.

**Table 1:**
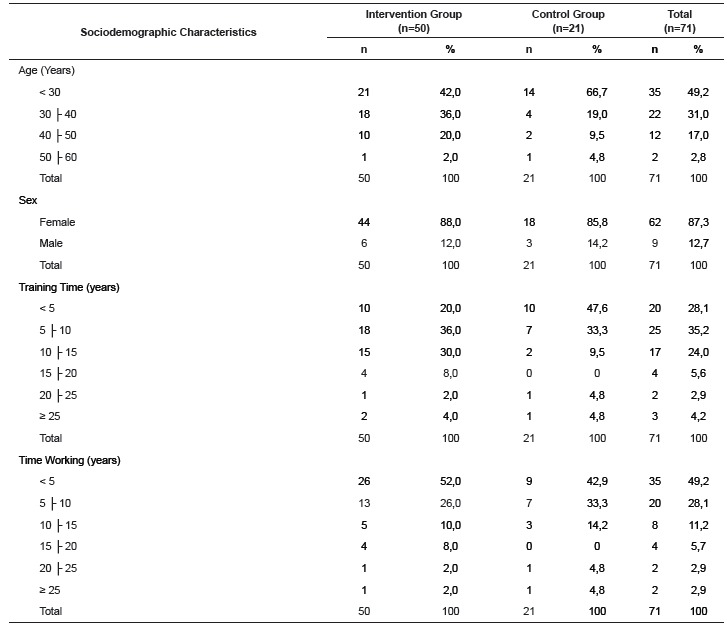
Distribution of frequencies and percentages of the groups according to socio-demographic characteristics. Vale do Rio Pardo e Jacuí region RS, Brasil, 2014

The intervention group and the control group showed similar socio-demographic characteristics. Regarding age, most of the professionals, 35 (49.2%) were in the age group below 30 years. The components of the intervention group had a mean of 33.8 years (SD* = 7.7 years) and the control group 30.5 (SD = 7.7 years). Regarding sex, we observed a higher frequency of women 62 (87.3%) in both groups. The intervention group showed greater training time (mean 9.0 years, SD = 5.7 years) than the control group (mean 6.7 years, SD = 7.1 years). However, the professional practice time is similar because the intervention group has an average of 6.7 years (SD = 6.1 years) and the control group 7.1 years (SD = 7.2 years).

In Table 2, the percentage of correct answers in the time before and after the intervention group and the control group regarding the staging items, evaluation and prevention of PU are expressed.

**Table 2:**
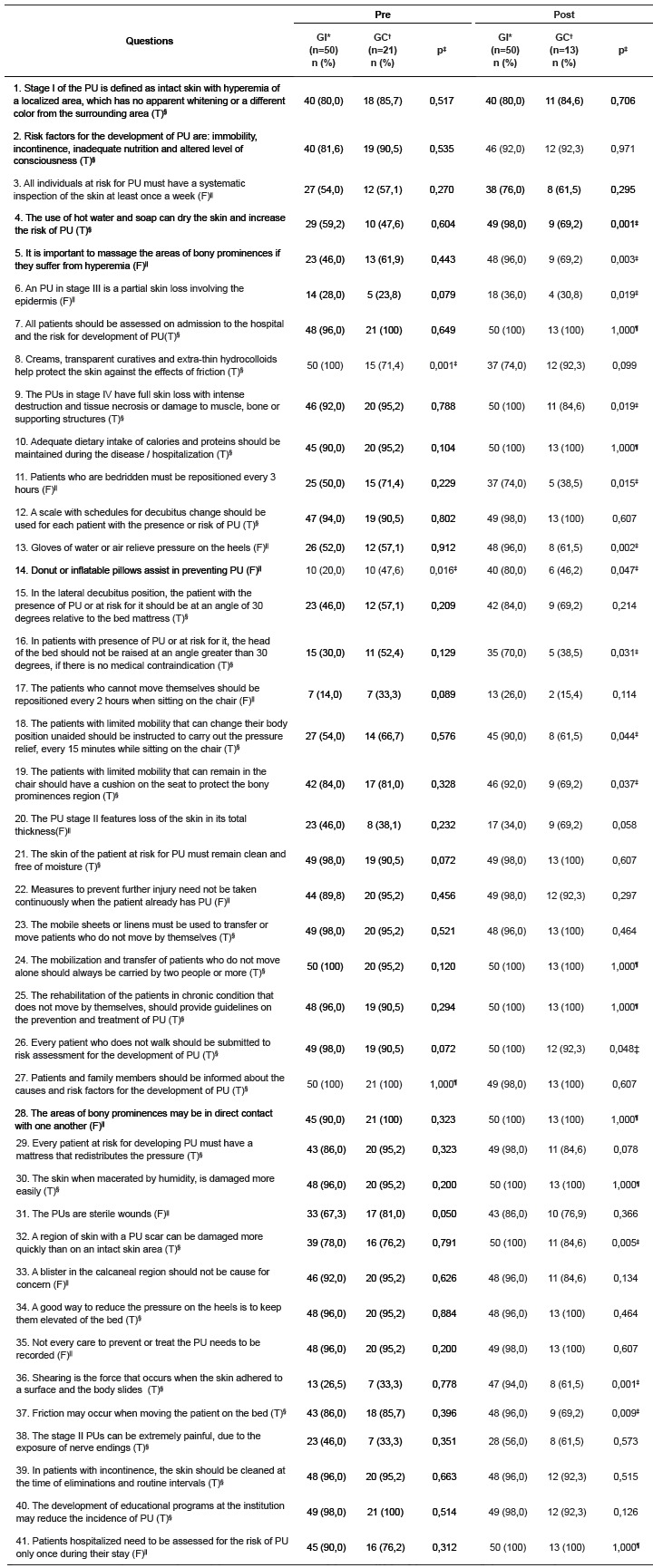
Distribution of the percentage of correct answers of the groups according to the questions about staging, assessment and prevention, pre and post educational intervention. *Vale do Rio Pardo e Jacuí* region, RS, Brasil, 2014

*IG = intervention group; †CG = control group ‡chi-square test, p<0,05; §(T) = true; ||(F) = false; ¶1,000 = all the answers with 100% accuracy

Two questions showed statistically significant difference when compared the questions between the intervention and control groups at the pre stage, while 14 questions showed differences at the post-stage (p<0,05).

The mean score of the groups, according to the knowledge test in the pre moment, was 74.1% (SD = 26.4) in the intervention group and 76.0% (SD = 22.9) in the control group, indicating no significant difference (p> 0.05) between groups. At the post moment, there was a significant difference (p = 0.001) between the groups. The intervention group averaged 87.8% correct answers (SD = 18.8), considering that the control group obtained 79.1% (SD = 22.2). Therefore, there is evidence of improvement in the mean score of the participants who received educational interventions, unlike the control group, which did not show a statistically significant improvement (p> 0.05).

The results for the correctness of the 41 questions of the knowledge test in the pre-intervention point, showed that the intervention group had 19 questions correct over 90% and the control group had 20 questions with over 90% accuracy. At the post moment the intervention group presented 29 questions over 90% correct, and the control group had 19 questions over 90% accuracy. The chi-square test showed that there was no relationship between gender and knowledge on PU (p> 0.05), age and knowledge on PU (p> 0.05), length of service and knowledge on PU (p> 0 , 05), and time of training and knowledge on PU (p> 0.05).

We identified the questions that the groups had lowest rate of correct answers, in the final knowledge test, which was below 70%. The lowest results of correct answers for the intervention group focused on four issues (number 6, 17, 20 and 38), one item concerning the prevention and three on staging UP. The control group had the loweest percentage of correct answers on 16 questions (number 3, 4, 5, 6, 11, 13, 14, 15, 16, 17, 18, 19, 20, 36, 37 and 38), 13 items related to prevention and three on staging. In the evaluation questions of PU (number 31 and 32), both groups had more than 75% accuracy.

## Discussion

The aim of this study was to compare the scores of knowledge about PU from the staff who participated in the educational intervention with the staff who did not attend the educational intervention. The results confirmed the hypothesis of the study, that professional nurses participating in educational intervention on PU have higher knowledge scores than those who do not participate. Although the intervention group did not reach the average of correct answers for the knowledge test (90%)^18^, the average value of 87.8% showing significant increase in correct responses score. The control group maintained a similar average scores in the pre and post stages, similar to the results of other national and international research, in which teams were evaluated with the same knowledge test without participating in educational programs, showed related average results^14^ ^,^ ^19^.

A similar result was found in a survey conducted in Brazil with nurses, auxiliaries and active nursing technicians in ICU. The Pieper knowledge test was used before and after the educational intervention. Nurses (n = 7) who participated only of pre-intervention phase and obtained 86.4% correct (SD = 4.6%), considering the assistants and nursing technicians in the pre-intervention phase (n = 25 ), they obtained 74.3% correct (SD = 14.8%) and in post intervention phase (n = 36), denoting improvement, with 81.2% correct (SD = 12.7%)^17^. The results showed improvement in the knowledge score of assistants and nursing technicians after educational interventions. However, the study did not have the adhesion of nurses in the educational program, which were not evaluated in the post point, making it impossible to analyze the educational program for these professionals. On the contrary, in the present study, all professionals in the intervention group participated in the interventions and responded to the initial and final testing, contributing to the proper evaluation of the educational program, minimizing possible bias in the study.

A similar study conducted in Jordan with 220 nurses from eight hospitals evaluated the effect of an educational program. The assessment of knowledge was done through a questionnaire, practice scales and test. The control and experimental groups reported no significant difference in pre-test scores. The experimental group, comparing the pre- and post-test, showed significant differences in scores on knowledge and practice (t = -5.47, p <0.001), attitudes (t = -6.8; p = 0 01) and intentions (t = -6.7; p <0.001). The authors concluded that the program has improved the knowledge, practices, attitudes and intentions on the prevention and treatment of PU in the experimental group^16^. The results mentioned corroborate this study, with the use of randomization and the use of the control group, which forms the basis for assessing the effect of the educational program.

According to the Pieper test, for a question to be considered as known, 90% of respondents should answer it correctly^18^. From the intervention group professionals evaluated in this study, 90% correctly answered 29 questions in the final questionnaire. A study conducted in Brazil, with the same test of knowledge, technicians and nursing assistants had more than 90% correct answers on 11 questions in the educational pre-intervention phase and 14 questions in the post-intervention phase. In the evaluation of authors, educational intervention contributed to the improvement in test results. However, they pointed out difficulties in the adherence of professionals to the program, which may have contributed to an average of more than 90% correct in only 14 questions after the intervention^17^.

At the end of the educational program of this research, the knowledge test showed the items in which the groups still need focus and elucidation. The intervention group needs to focus on issues relating to the description of the stages of PU and repositioning the patient when sitting. On the other hand, the control group requires a more general education, including the description of the stages and prevention of PU. These data confirm the national study results conducted with nursing teams, in which the pre-intervention point showed that the lowest percentage of correct answers was on issues relating to the description of stages II and III of PU and repositioning the patient when sitting. During the post intervention moment, a knowledge deficit remained on these issues^17^. Identifying these deficient areas can guide the planning strategies for the dissemination and adoption of appropriate preventive measures by the teams^14^ ^,^ ^20^.

In this research, the socio-demographic variables showed no relationship with knowledge on UP. The analysis was carried out considering the professional category, nurses and nursing technicians separately, which may explain the lack of association. The study results are similar to a study that evaluated the knowledge of 228 nurses in the United States on the prevention and staging of UP. The study revealed that knowledge of these professionals was not related to age, educational level and years of professional experience^18^.

The limitations of this study relate to the methodology of the educational program used as a specific approach, without reapplying the knowledge test in the third moment to see if there was consolidation of knowledge. Researchers in New Zealand have evaluated the impact of an educational program offered to the ICU nurses, they were measured at three times: before the educational intervention two weeks and 20 weeks later. The results showed significant difference in the scores obtained in the pre-intervention period and in the second week post-intervention. However, there was no difference in the comparison of scores between the pre-intervention period and after 20 weeks. The authors concluded that the knowledge of nurses improved with the educational program, but soon returned to the baseline level^21^. These results highlight the importance of the continued education activities on PU with nursing professionals^9^ ^,^ ^14^ ^,^ ^16^ ^-^ ^17^. Therefore, it is suggested to conduct further research concerning the subject, with experimental design and control group, measuring medium and long-term knowledge of the teams, as well as seeking to understand personal and institutional factors that can be obstacles for the prevention of PU.

As a practical application of this study, it is possible to mention the positive effects of educational interventions identified by the incidence data collected in the ICU of the intervention group. Four months before the educational intervention, the incidence was 17.2% of PU and after four months of educational intervention, it was found incidence of 14.2%. Similar results were validated in a recent survey conducted in Brazil, in which the authors observed decreased incidence of PU in ICU after the implementation of a risk assessment protocol and prevention of PU^9^.

There is no consensus among authors about the ideal time interval for educational interventions. Researchers^22^, warn about the importance of the continued education and the implementation of PU prevention programs in interdisciplinary team work 24 hours a day. The authors advise to pay constant attention to the process and conclude that the incidence of PU increases again when PU prevention programs are ignored.

The implication for the practice of intervention in this study is related to overload in the ICU environment, contributing to long periods when the researcher was waiting for the staff to perform the educational intervention. In the current study, researchers suggest that there should be less workload so that professionals can participate in educational interventions, thus contributing to the improvement of the continued education process within the ICU^23^.

A positive aspect in this study to be revealed is the feedback from the favorable perception of interventions and educational program, as well as the interest in the improvement of the participants who were motivated showing a rate of 100% adherence to the program. These data corroborate the results of a similar survey, which used the feedback in the evaluation of the intervention and the continued education program, noting positive influence on performance and professional qualification^24^.

## Conclusion

The educational interventions on staging, evaluation and prevention of PU contributed to the increase in the correct answers score on the knowledge test in the intervention group and improved knowledge on the subject. The strategies used to operationalize the educational intervention performed in this study are important means for the dissemination of recommendations for the prevention of PU. They can be used by nursing managers, policy makers, educators and researchers as facilitating strategies for the development and implementation of educational programs for the prevention of PU.
